# Progress in Phototherapy and Electro-Therapeutics

**Published:** 1903-10-10

**Authors:** 


### Progress in Phototherapy and Electro-therapeutics.

(*Continued from page* 19.)

Pusey [25] tells of an enormously extensive cutaneous cancer with epitheliomatous ulcers in the neck, extending from the spine of the scapula to below the clavicle in front. Complete extirpation could not be performed, so *x*-rays were used on January 15th and continued daily till April 16th when all the ulcers were healed except a small one which took till May 8th to disappear. The patient was completely relieved at the date of report.

A case of Fisk's is reported by Johnson [26] where an old man æt. 70 had had six operations for a recurrent round-celled sarcoma of the neck; the erysipelas toxins had been used and the external carotid had been tied without success. *X*-rays were applied in December, 1901, and in six weeks the entire growth had disappeared. Subsequently metastases occurred in the axilla and groins, but these yielded to the rays. In January, 1903, he was free from recurrence. Johnson, as also Morton [27] and Robinson,[28] consider that the rays should be used before and after operation.

In the study of rodent ulcers, Sequeira [29] finds evidence from the character of the cells in some sections that the disease may begin in the hair follicle; but there is also evidence that the disease may begin in the sebaceous gland. After the rays the nuclei become disorganised and the cell plasm breaks up, often becoming fatty. Recurrences seem to be due to cells escaping the action of the rays. There are cells found in the sections quite unchanged, taken from apparently cured cases, yet some have remained without recurrence for three years. He has treated 100 cases; two cases were rebellious to the treatment. There was no doubt in the diagnosis of one of these, as it was examined microscopically. The second also diagnosed microscopically, partially healed and spread at the same time. Where cartilage is involved with the skin, the contraction necessary for healing cannot take place as in the case of an ulcer involving the tibia. There are slight recurrences in nearly half of all the cases, and in some more than once. As a rule these recurrences are easily dealt with by fresh applications; *x*-rays are the best therapeutic agent for rodent ulcer where complete excision cannot be carried out. In one case the condition began at the age of 12, which is, he thinks, the earliest case on record.

In the opinions of Malcolm Morris and Dore [30] the *x*-rays are not bactericidal, but they stimulate the growth of bacteria, so that the good results obtained are not due to the action on bacteria. They may be due to the corrosive action of minute platinum particles, to ozone generation (Tesla), electric waves given off from the tube (Schiff), or retarded osmosis. In lupus the secondary conditions, *e.g.*, ulceration, discharge, œdema, etc., are relieved at once, but in respect of the eradication of disease, progress is disappointingly slow and failure is not infrequent. They proved of no use in one case of ulceration of the palate. It is the epidermic cells, more especially in the hair follicles, which react so readily to strong stimulation, not the underlying connective tissue. Deep ulcerated cases offer less risk of dermatitis. In carcinoma the *x*-rays relieve pain and may destroy secondary nodules as they appear. Inflammatory reactions are harmful and the exposures should be weak, especially in acute inflammatory conditions. In keloid they diminish the growth and ease pain. They have had good results in acne rosacea, eczema, psoriasis, hypertrichosis, sycosis, ringworm, favus and mycosis fungoides. In lupus, though a useful addition, the rays are much inferior to the light in curative efficacy.

As an adjunct to the *x*-ray treatment of lupus Hall Edwards [31] recommends the use of a saturated solution of permanganate of potash. He prepares the part with ichthyol or carbolic soap and wipes with a pledget moistened with methylated spirit. When dry he paints on the solution with a brush. Crusts are not removed but are saturated with the solution, applications being made daily or every other day. This plan gives good results also apart from the rays.. He urges that this is a better method than using a 2 per cent. solution as suggested by Butte applied 12–15 minutes daily, or than the dry powder spread on the part with a spatula : this latter being severe and producing a considerable amount of inflammation. The best results have been obtained in those cases where under the *x*-rays the centres of the patches get well while the edges spread. In those where the centres have cicatrized and the edges are covered with dry crusts two applications have been sufficient to cause the crusts to drop off, leaving behind a dry and healthy scar : the active parts remaining require frequent treatment. This treatment has little effect on the indurated shiny, non-ulcerated, slow-growing patches which are also so difficult to treat with light or *x*-rays. No staining is left.

The list of diseases which may be improved by *x*-ray treatment is growing gradually. Zeisler [32] has treated cases of lupus vulgaris and erythematosus, scrofuloderma, hypertrichosis, sycosis, acne (34 cases) ; epithelioma, psoriasis, lichen planus hypertrophicus, dermatitis staphylogenes, eczema, keratosis palmaris, clavus, pruritus, and hyperidrosis nasi. He is pleased with the results obtained in all, but especially so in lupus vulgaris, sycosis, hypertrichosis, and acne of all kinds. In the latter the benefit was observed early in the treatment, and was very pronounced. Acne rosacea, acne necroticans, the indurated, the pustular form, and the ordinary acne of the face are included in those treated. Some of the severest cases were cured in from four to six weeks. In lichen planus hypertrophicus a patch which had resisted energetic treatment, such as curetting, disappeared after four strong exposures given at intervals of five days, leaving the skin in a perfectly normal condition. In isolated, chronic, indurated patches of eczema the result is very encouraging. Keratosis palmaris and clavus have yielded to the rays. In a case of xeroderma pigmentosum in a child aged five, after some of the lesions had disappeared under thyroid extract, one was left which resisted treatment but did not improve under the *x*-rays, though marked dermatitis had supervened.

Mycosis fungoides has yielded to the rays in the hands of Dr. Stainer,[33] the result being that the patient is apparently free from the disease. Sydney Stephenson [34] has had success with the rays in the treatment of a case of conjunctival tuberculosis in a child aged four. Nine exposures were given at 6 to 10 inches distance, on the average each exposure lasting for 10 minutes, between December 2nd and January 1st. Four more were given on February 21st and 28th, March 5th and 10th, and by April 3rd the general condition was excellent ; the left eye, which was the affected one, was as widely open as its fellow, and the conjunctiva seemed wholly free from the disease. The only cicatrices present were those produced by the removal of material for diagnostic purposes.

The results of chronic exposure of the skin to the rays from high vacuum tubes are described by Prince.[35] At first the hand was red and swollen from the wrist to the finger-tips, without loss of hair or desquamation. This subsided spontaneously but occurred five months later, and persisted, with brittleness of nails and desquamation, and the dorsum of the fingers remained swollen. The redness was most marked in warm weather. Warty growths formed on the fingers and the nail-roots became fibrous. The senses of touch and pain were impaired, those of heat and cold were normal. The condition seems to be one of disturbance of the nutrition of the skin with chronic fibrosis of the derma. The best application was found to be ol. morrhuae, zinc oxide and lanoline. Schmidt [36] gives a peculiar case of marked atrophy of the skin of the hand, resulting from half an hour's exposure during the taking of a skiagram. Two or three weeks after the exposure the skin became red, then bluish, and then became like wrinkled cigarette paper. There was no œdema or vesiculation. The atrophy has remained permanent for five years.

Another use of the rays is suggested by James Cameron,[37] who found a skiagram useful as confirmatory evidence as to the age of a child at birth, when he was required to give such evidence in a court of law. The skiagram showed the absence of the lower femoral centre of ossification, and the presence of those of the os calcis and astragalus respectively. The sternal centres were obscured by the heart shadow.

[25] Brit. Med. Jour., June 6. [26] Ibid. [27] Med. Rec., May 30. [28] Brit. Med. Jour., June 6. [29] Ibid. [30] Ibid. [31] Ibid., June 27. [32] Ibid., June 13. [33] Lancet, May 23. [34] Brit. Med. Jour., June 6. [35] Brit. Jour. of Dermatol., May. [36] Ibid., June. [37] Brit. Med. Jour., May 23.

(*To be continued.*)

